# Plant responses to flooding

**DOI:** 10.3389/fpls.2014.00226

**Published:** 2014-05-23

**Authors:** Chiara Pucciariello, Laurentius A. C. J. Voesenek, Pierdomenico Perata, Rashmi Sasidharan

**Affiliations:** ^1^Institute of Life Sciences, Scuola Superiore Sant'AnnaPisa, Italy; ^2^Plant Ecophysiology, Institute of Environmental Biology, Utrecht UniversityUtrecht, Netherlands

**Keywords:** anoxia, flooding, hypoxia, low oxygen, submergence, waterlogging

Climate change models predict an increase in the frequency of flooding events globally, making flooding stress a major environmental threat for plants. Annually, crop damages due to unseasonal and severe flooding events amount to billions of dollars in yield losses. Despite the vulnerability of most crops to wet conditions, there is significant variation in the plant tolerance to flooding. Plant species adapted to wet areas have evolved specific strategies to deal with and even thrive under these conditions. In order to generate flood resilient, high yielding crops, it is of essence to not only understand the different elements that define flooding stress, but also to determine how plants sense and respond to these signals. In addition, the understanding of the genetic basis of tolerance variation and the underlying genes and processes will be key to discovering novel tolerance mechanisms and ultimately translating these to crops. In the last few years, the flooding research community has made leaps and bounds in understanding several of these aspects, unraveling more layers of the plant flooding response and paving the way for further research. This special issue entitled “Plant responses to flooding stress” brings together a collection of review and original research articles reflecting the broad scope and dynamics of the field of plant anaerobiosis.

The conditions during flooding can drastically affect survival. Large survival differences exist between plants submerged in complete darkness versus those submerged with some light, hinting at the importance of underwater photosynthesis. This importance is not only related to the production of carbohydrates but also, to the generation of molecular oxygen that accumulates in submerged plants and diffuses to tissues with less oxygen (e.g., roots). Pedersen et al. (2013) summarize recent advances and methods to quantify underwater photosynthesis in terrestrial plants in relation to leaf acclimations.

Among cereals, rice has the unique capacity to germinate and grow vigorously in flood-prone areas. Rice adaptive plasticity to different hydrological regimes has allowed the selection and characterization of some genotypes that have been subsequently used to breed high yielding and tolerant modern varieties. Miro and Ismail (2013) present an overview of the current understanding of the mechanisms associated with tolerant traits such as anaerobic germination and early vigor in rice that can help to develop tolerant varieties for direct-seeding systems. Narsai and Whelan (2013) employed a meta-analyses of microarray data to compare global low oxygen transcriptomic responses of tolerant rice with the relatively intolerant Arabidopsis. Based on their results they conclude that while Arabidopsis is simply responding to general stress conditions, rice displays transcriptome reconfigurations specific to low oxygen conditions.

Energy limitation during hypoxia, necessitates several resource limiting measures in plants. Amongst these is the use of pyrophosphate (PPi)-dependent enzymes that can take PPi as a substrate to catalyze reactions, thus sparing precious ATP reserves. Mustroph et al. (2013), execute a detailed expression analyses on the rice ATP and PPi-dependent phosphofructokinase gene family to establish their role during anoxic stress.

The study of flood adaptive strategies that evolved in nature and were selected by farmers will be crucial to develop stress-tolerant crop varieties. In this context, other environmental factors likely influence submergence tolerance, and stress combinations need to be explored toward the selection of crops tolerant to multiple stresses. Zeng et al. (2013) studied the combined response to waterlogging and salinity of barley varieties. They conclude that hypoxia is not the only factor determining the differential response of the genotypes to the stress combination and that the soil type strongly influence the presence of elemental toxicity.

The accumulation of toxic substances in flooded soils is a serious concern for plants. In some plant species, the formation of an apoplastic suberin barrier in the roots can help to prevent toxins entry and at the same time loss of oxygen. Watanabe et al. (2013) review the role of this barrier in waterlogging tolerance. In a similar context, Lamers et al. (2013) provide an overview of the effect of toxic sulfide accumulation in fresh and marine water. The accumulation of sulfide by microbial activity occurring in aquatic ecosystems is exacerbated by anthropogenic inputs. This makes sulfide-related pollution an urgent question to address.

Reactive oxygen species (ROS) are a major component of low oxygen stress. ROS production is expected to contribute to submergence adaptation. Owing to their transient nature ROS detection has been famously difficult to accurately quantitate. Steffens et al. (2013) outline the role of ROS during flooding and review the potential of techniques such as electron paramagnetic resonance to investigate ROS *in planta*.

Large trees encounter specific problems such as relatively deep floods (10–15 m) and prolonged durations of these floods (up to 7 months). Herrera (2013) reviews the biochemical responses of tropical trees to flooding in South America. The emphasis in this overview lies on leaf gas exchange, stomatal conductance, water status, and carbohydrate balance.

Beside plants, the unicellular green alga *Chlamydomonas reinhardtii* has received special attention for its response to low oxygen due to its capacity to produce H_2_, a source of renewable energy, under anoxia. Catalanotti et al. (2013) describe the fermentation pathway of *Chlamydomonas*, also discussing the fermentation process evolution.

This Frontiers special showcases the broad scope and importance of low oxygen stress studies in plants. We are grateful to all the authors for contributing to this collection. We would also like to acknowledge all the reviewers for taking the time to assess the work submitted here and helping to shape this focus issue.

## Conflict of interest statement

The authors declare that the research was conducted in the absence of any commercial or financial relationships that could be construed as a potential conflict of interest.
